# An *in silico* bioinformatics framework for prioritizing candidate genes in the coronary no-reflow phenomenon

**DOI:** 10.21542/gcsp.2026.14

**Published:** 2026-04-30

**Authors:** Fadhlan Abdur Rahman, Suryono Suryono, Aditha Satria Maulana, Pipiet Wulandari

**Affiliations:** 1 Citra Husada Hospital, Jl. Teratai No. 22, Gebang, Patrang, Jember, 68117, East Java, Indonesia; 2 Department of Cardiology and Vascular Medicine,dr. Soebandi Regional General Hospital, Jl. dr. Soebandi No. 124, Patrang, Jember, 68111, East Java, Indonesia; 3 Faculty of Medicine, University of Jember, Jl. Kalimantan No. 37, Kampus Tegalboto, Jember, 68121, East Java, Indonesia

## Abstract

**Background:** The coronary no-reflow phenomenon remains a major obstacle to optimal outcomes after primary percutaneous coronary intervention. Its mechanisms are multifactorial and not fully clarified, with genetic components increasingly recognized as important contributors. This study aims to identify key genes, characterize their biological functions, and explore *in silico*–based therapeutic options targeting genes implicated in this condition.

**Methods:**Genes related to the coronary no-reflow phenomenon were retrieved from the GeneCards database. A protein–protein interaction network was constructed using STRING, and top-ranked genes were identified via the maximal cliques centrality and maximum neighborhood component algorithms in Cytoscape. Gene ontology enrichment analysis was performed using WebGestaltR. Three-dimensional protein structures were generated with SWISS-Model and validated using ERRAT and PROCHECK.

**Results:** Seventy-nine genes with significant relevance were identified. Network analysis yielded 55 nodes, from which eight hub genes were identified: IL6, TNF, ICAM1, MPO, ALB, MMP9, CXCL8, and CRP. Enrichment analysis highlighted blood vessel diameter maintenance and coagulation as key biological processes, with antioxidant activity as the leading molecular function. MPO, CRP, CXCL8, and ALB demonstrated favorable structural characteristics, supporting their prioritization.

**Conclusion:** MPO, CRP, CXCL8, and ALB are central genes in the coronary no-reflow phenomenon and warrant further experimental validation for therapeutic development.

## Introduction

Acute myocardial infarction remains one of the leading causes of mortality worldwide. Primary percutaneous coronary intervention (PCI) is the current gold-standard reperfusion strategy for patients with ST-elevation myocardial infarction (STEMI). However, successful restoration of epicardial coronary artery patency does not always translate into adequate myocardial perfusion. One frequently underrecognized complication is the coronary no-reflow (CNR) phenomenon, a condition in which myocardial tissue perfusion remains insufficient despite the absence of residual epicardial obstruction.

CNR occurs in approximately 4–7% of patients with acute coronary syndrome and is associated with a significant increase in mortality and major adverse cardiac events, reaching up to 20%^1,2^. The underlying mechanisms are multifactorial, including delayed reperfusion, intervention in saphenous vein grafts, involvement of the left anterior descending artery, high thrombus burden, excessive balloon inflation pressure, and the use of devices such as rotational atherectomy^3^. Beyond these procedural factors, emerging evidence suggests that individual susceptibility to no-reflow may also be influenced by intrinsic biological factors, including genetic predisposition.

Despite growing recognition of procedural and clinical predictors, the molecular and genetic mechanisms underlying CNR remain incompletely characterized. In particular, an integrated bioinformatics approach to systematically identify and prioritize key genes involved in CNR is still lacking. Given these considerations, the present study aims to identify critical genes associated with the no-reflow phenomenon and to develop an in silico-based exploratory framework for candidate targets for further investigation.

## Methods

### Genetic data acquisition

Genes associated with the coronary no-reflow phenomenon were retrieved using the GeneCards database (https://www.genecards.org). Genes ranked by relevance score were selected and downloaded for further analysis, resulting in a total of 79 genes included in the subsequent analyses (Table S1)^4^. This study followed a stepwise bioinformatics pipeline, beginning with gene retrieval from GeneCards (*n* = 79), followed by protein–protein interaction filtering (*n* = 55), and hub gene prioritization (*n* = 8).

### Functional annotation and pathway enrichment analysis

Functional enrichment analysis was performed using the Kyoto Encyclopedia of Genes and Genomes (KEGG) pathways and Gene Ontology (GO) terms. Overrepresentation Enrichment Analysis (ORA) was conducted via WebGestalt 2019 (http://www.webgestalt.org)^5^. A false discovery rate (FDR) <0.05 was applied as the threshold for statistical significance.

### Construction of the protein–protein interaction (ppi) network

The protein–protein interaction (PPI) network was constructed using the STRING database v11.0b (https://string-db.org) with a high-confidence interaction score threshold (>0.7)^6^. The curated gene set was input into STRING, and only genes with recognized interactions under this confidence criterion were retained.

As a result, 55 genes were included in the final PPI network. The resulting network was then imported into Cytoscape for visualization and further analysis^7^. Key hub genes were identified using the CytoHubba plugin in Cytoscape. Two topological algorithms, maximal clique centrality (MCC) and maximum neighborhood component (MNC) were applied independently. The top-ranked genes from each method were compared, and overlapping genes were selected as key hub genes for further structural and functional investigation.

### Protein structure modeling

Three-dimensional structural models of the proteins encoded by the top ten hub genes were generated using the SWISS-MODEL server (https://swissmodel.expasy.org). Protein sequences were obtained from the NCBI RefSeq Protein Database in FASTA format. The modeling pipeline selected structural templates from the Protein Data Bank (PDB) based on the best available Global Model Quality Estimate (GMQE) and QMEAN scores^8^. When available, experimentally resolved structures in the Protein Data Bank were considered as primary templates; otherwise, homology modeling was performed based on the best available templates.

### Protein structure validation

The quality of the predicted protein models was assessed using SAVES v6.0 (UCLA–DOE Institute for Genomics and Proteomics), which integrates several structure evaluation tools:

• **ERRAT** was used to evaluate the overall model quality based on non-bonded atomic interactions. Models with an ERRAT score >80% were considered structurally reliable.
• Stereochemical validation was performed using **PROCHECK**, which analyzes backbone dihedral angles through the Ramachandran plot. Models with >90% of residues in the “most favored” and “allowed” regions were considered structurally stable and suitable for downstream applications, including molecular docking.

## Results

### Gene identification related to no-reflow

A keyword-based search of the GeneCards database using *“no-reflow phenomenon”* identified 79 genes associated with coronary no-reflow (CNR) (Table S1). Following duplicate removal and verification, the curated gene set was subjected to subsequent systems-level analyses to characterize functional relationships and underlying biological mechanisms.

### Functional enrichment analysis

**Figure 1.**
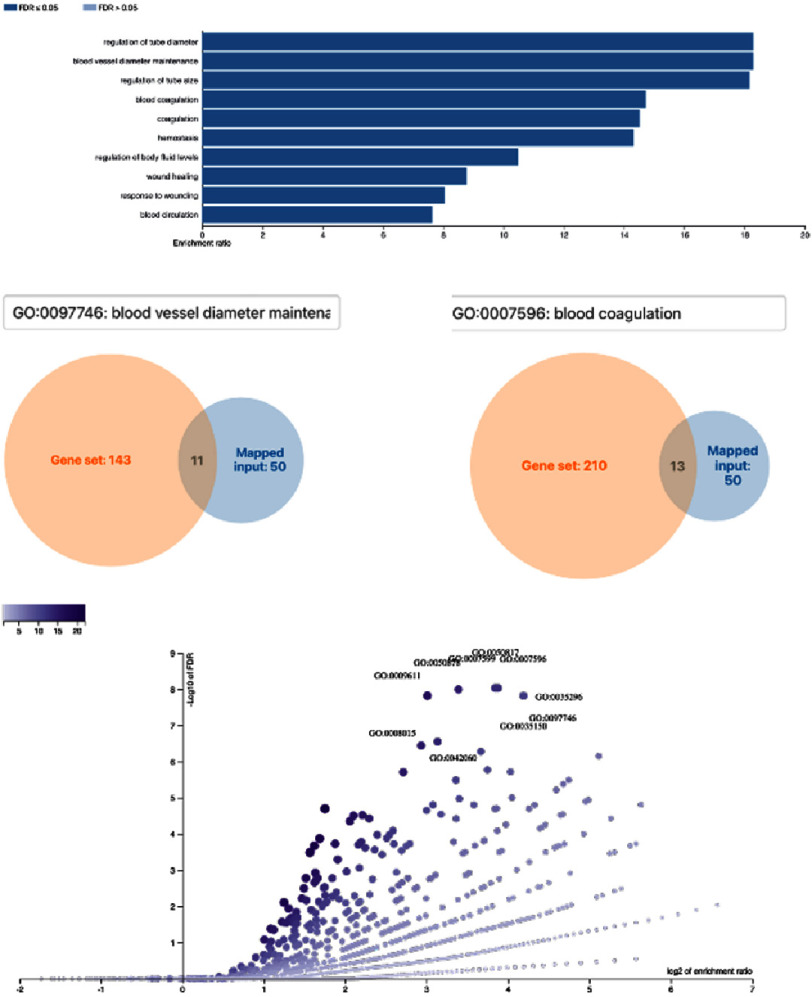
Functional enrichment analysis showed 10 biological process of genes related to no-reflow phenomenon FDR value (*p* <0.05). There were also 11 genes which are the most corresponding to the “blood vessel diameter maintenance” as the most significant biological process, and 13 genes which are the most corresponding to the “blood coagulation” as significant biological process involved in no-reflow phenomenon.

**Figure 2.**
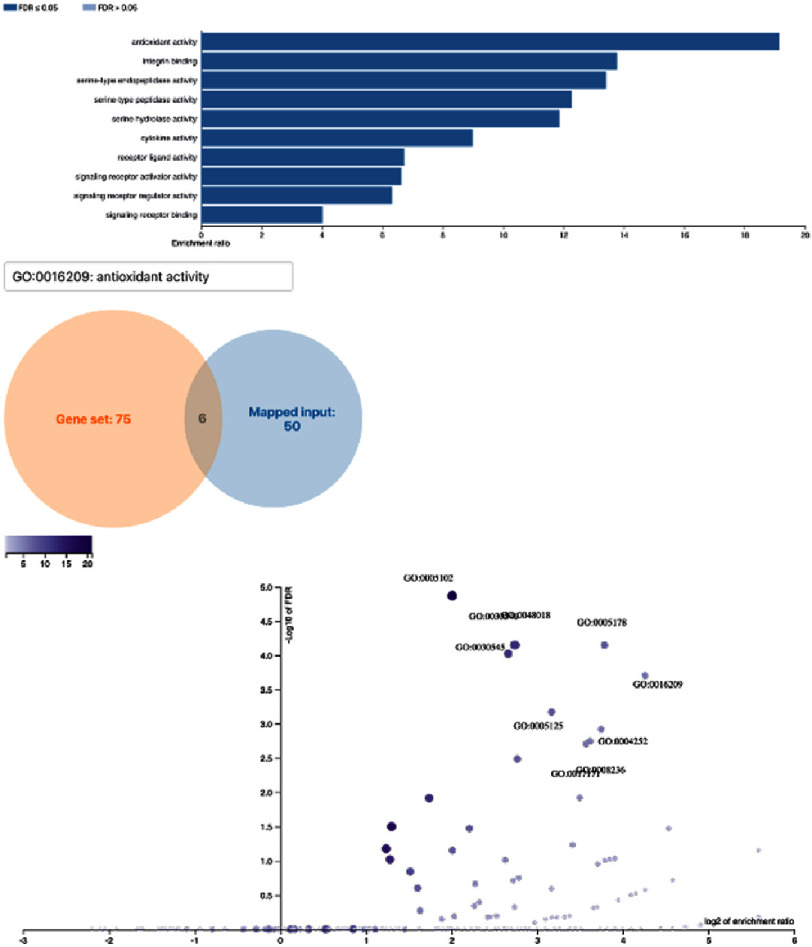
Functional enrichment / gene ontology analysis (molecular function). Functional enrichment analysis showed 10 molecular functions of genes related to no-reflow phenomenon FDR value (*p* <0.05). There were also 6 genes which are the most corresponding to the “antioxidant activity” as the most significant molecular function involved in no-reflow phenomenon.

GO and KEGG enrichment analyses performed via WebGestalt identified ten significantly enriched biological processes (FDR < 0.05). The most significant process was blood vessel diameter maintenance (11 genes), followed by blood coagulation (13 genes), highlighting the involvement of vascular tone regulation and hemostatic activity (Figure 1). Ten significantly enriched molecular function categories were also identified, reflecting roles in inflammatory mediator activity, oxidative stress regulation, ligand receptor interaction, and enzymatic modulation (Figure 2). Antioxidant activity emerged as the most significantly enriched molecular function among CNR-related genes.

### Protein–protein interaction network analysis

**Figure 3.**
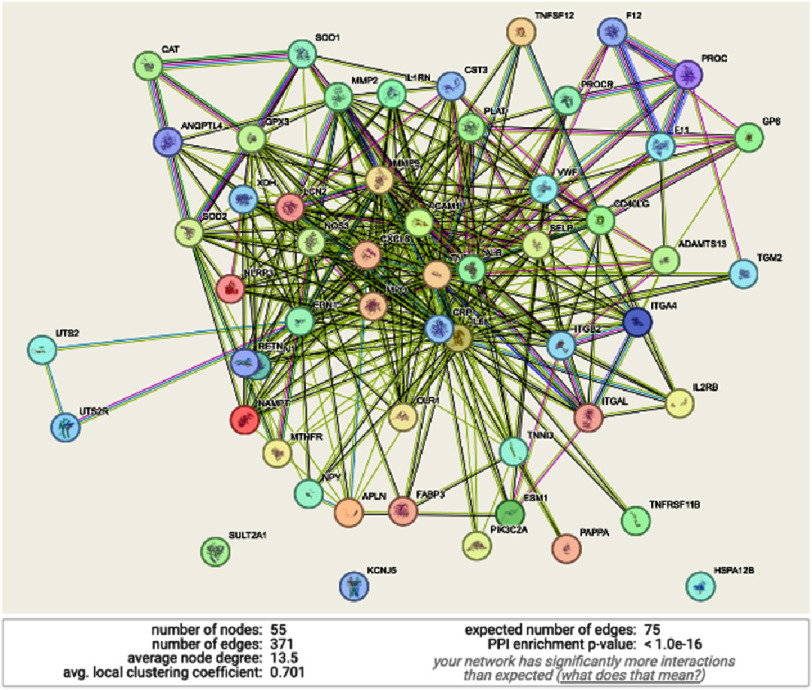
Protein-protein interaction network (PPIN) analysis. PPIN analysis using STRING tools of the hub-genes (55 genes with high relevance score) related to no-reflow phenomenon with PPI enrichment *p*-value showed the significant value (<1.0e−16), indicating that the observed number of edge is significant, the nodes are not random, and the genes are related to each other.

A total of 79 genes were initially identified from the GeneCards database and included for downstream analysis. Following application of a high-confidence interaction threshold in STRING (>0.7), 55 genes were retained in the final PPI network. STRING network analysis demonstrated a highly interconnected PPI network consisting of 55 nodes and 75 edges (PPI enrichment *p*-value < 1.0 ×10^−^^16^), indicating coordinated biological activity rather than random interactions (Figure 3).

**Figure 4.**
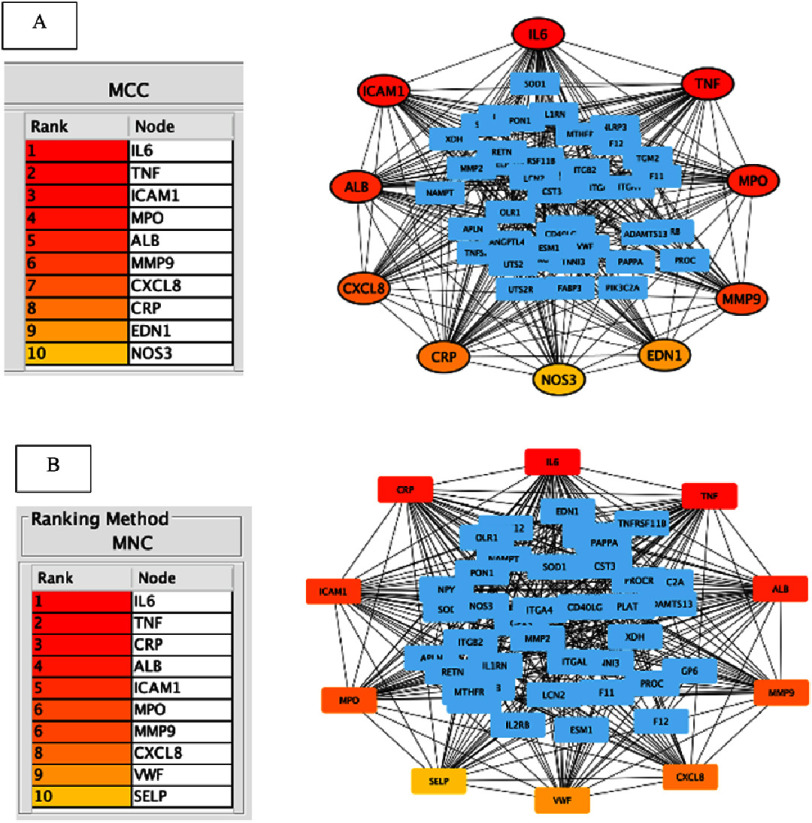
Hub gene analysis. Hub gene analysis was performed using the PPI network derived from 55 genes with high-confidence interactions. The CytoHubba plugin was applied using maximal clique centrality (MCC) (Figure 4A) and maximum neighborhood component (MNC) algorithms (Figure 4B). Each approach gives the different top 10 genes (colored by red, orange, and yellow), **with the red color indicating the higher potential genes that contribute to no-reflow phenomenon.** From those two approaches, there were some intersection genes that are IL6, TNF, ICAM1, MPO, ALB, MMP9, CXCL8, and CRP which are the most significant and contributing to biological process and molecular function involved in no-reflow phenomenon.

Centrality analyses using CytoHubba revealed essential hub genes across different functional domains. MCC analysis identified IL6, TNF, ICAM1, MMP9, and CXCL8 as core regulators within inflammatory and endothelial dysfunction pathways, while MNC analysis highlighted VWF and SELP as key connectivity hubs involved in platelet activation and endothelial adhesion processes. The intersection of MCC and MNC analyses resulted in eight overlapping hub genes: IL6, TNF, ICAM1, MPO, ALB, MMP9, CXCL8, and CRP (Figure 4).

### Protein structure validation of candidate genes

**Figure 5.**
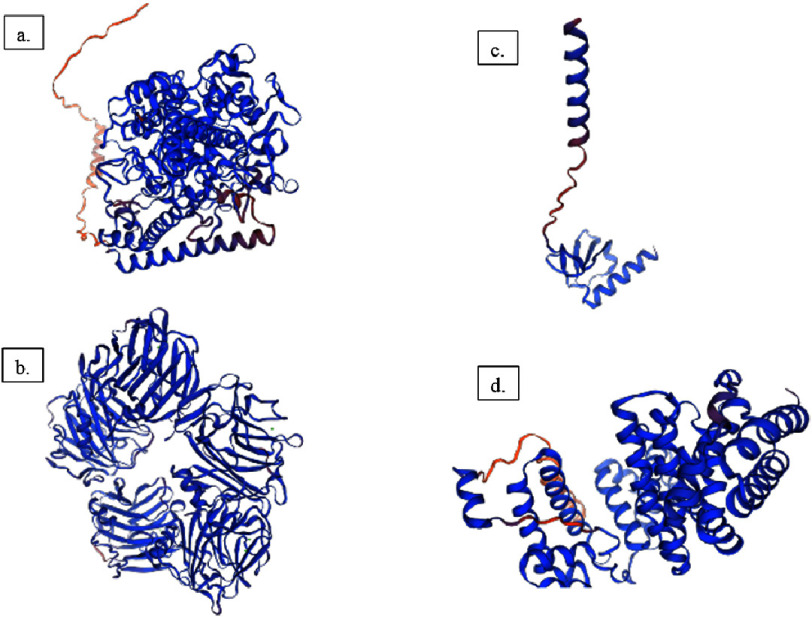
Construction of 3D protein structure of MPO (a), CRP (b), CXCL8 (c), and ALB (d).

Eight hub genes were further validated at the protein level. Protein sequences retrieved from the NCBI RefSeq database (FASTA format) were subjected to 3D structural modeling using SWISS-MODEL (Figure 5). Model quality was assessed using ERRAT and PROCHECK (Ramachandran plot). The quality scores were as follows:

• **IL-6:** ERRAT 87.5%; PROCHECK 93% of residues in allowed regions
• **TNF:** ERRAT 86.3%; PROCHECK 85.2%
• **ICAM1:** ERRAT 83.7%; PROCHECK 91%
• **MPO:** ERRAT 97.0%; PROCHECK 90.7%
• **ALB:** ERRAT 89.9%; PROCHECK 94.1%
• **MMP9:** excluded due to low model coverage (80.9%)
• **CXCL8:** ERRAT 93.2%; PROCHECK 89.9%
• **CRP:** ERRAT 92.3%; PROCHECK 90.2%

**Figure 6.**
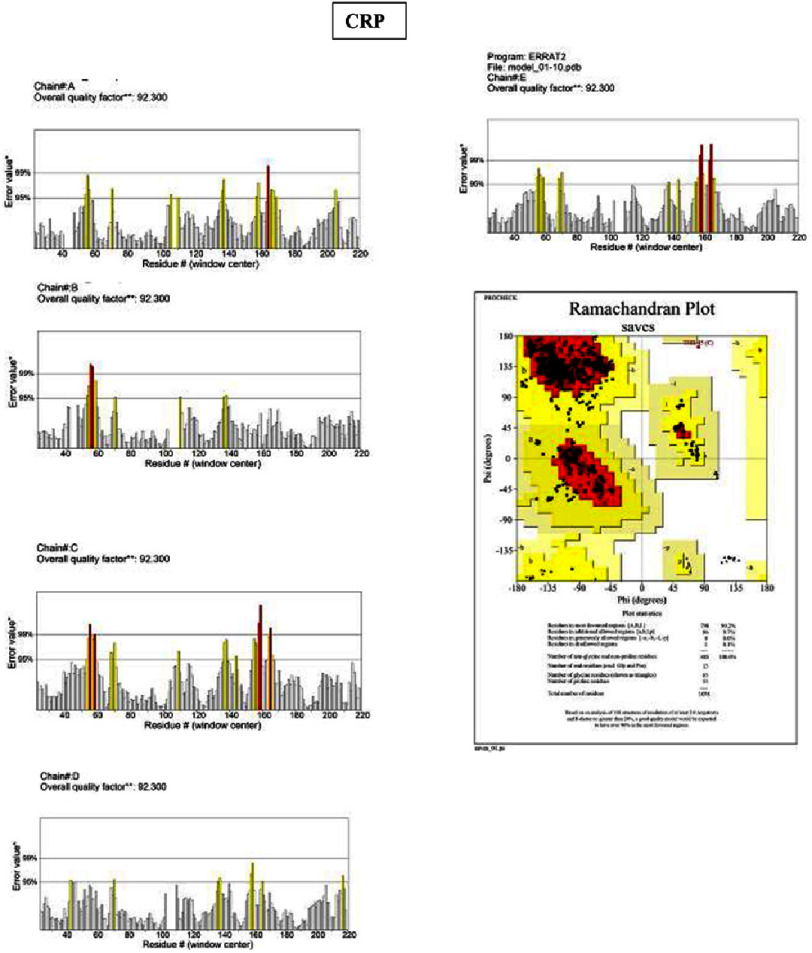
ERRAT-PROCHECK validation analysis. Analysis using UCLA-DOE LAB—SAVES v6.1 tools. ERRAT analysis showed a standard overall quality factor value CRP (92.3%) indicating good structural quality and suitability for further structural and functional analysis. A PROCHECK analysis of the Ramachandran plot revealed the high value of residues in the allowed region of the CRP (90.2%), indicating good stereochemical quality and structural stability of the protein model.

**Figure 7.**
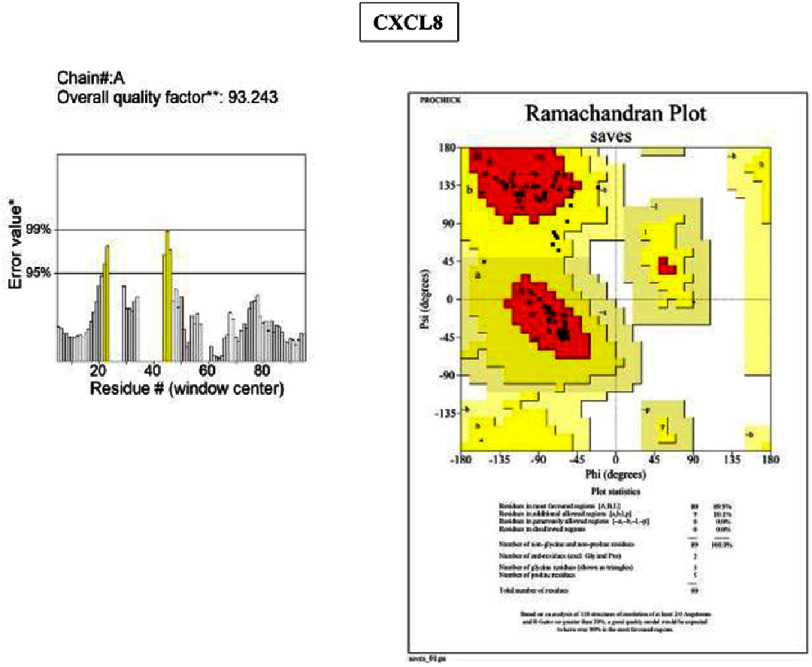
ERRAT-PROCHECK validation analysis. Analysis using UCLA-DOE LAB—SAVES v6.1 tools. ERRAT analysis showed a standard overall quality factor value CXCL8 (93.2%) indicating good structural quality and suitability for further structural and functional analysis. A PROCHECK analysis of the Ramachandran plot revealed the high value of residues in the allowed region of the CXCL8 (89.9%), indicating good stereochemical quality and structural stability of the protein model.

**Figure 8.**
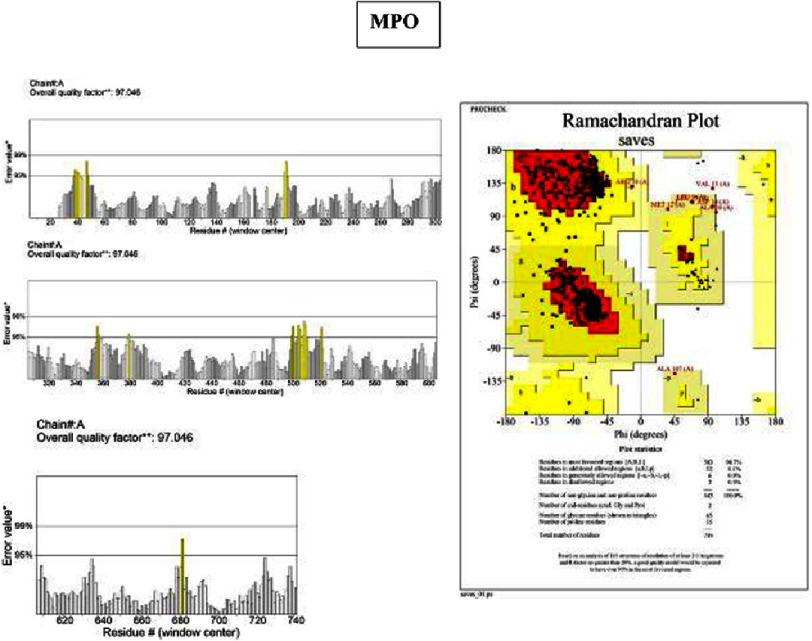
ERRAT-PROCHECK validation analysis. Analysis using UCLA-DOE LAB—SAVES v6.1 tools. ERRAT analysis showed a standard overall quality factor value MPO (97%) indicating good structural quality and suitability for further structural and functional analysis. A PROCHECK analysis of the Ramachandran plot revealed the high value of residues in the allowed region of the MPO (90.7%), indicating good stereochemical quality and structural stability of the protein model.

**Figure 9.**
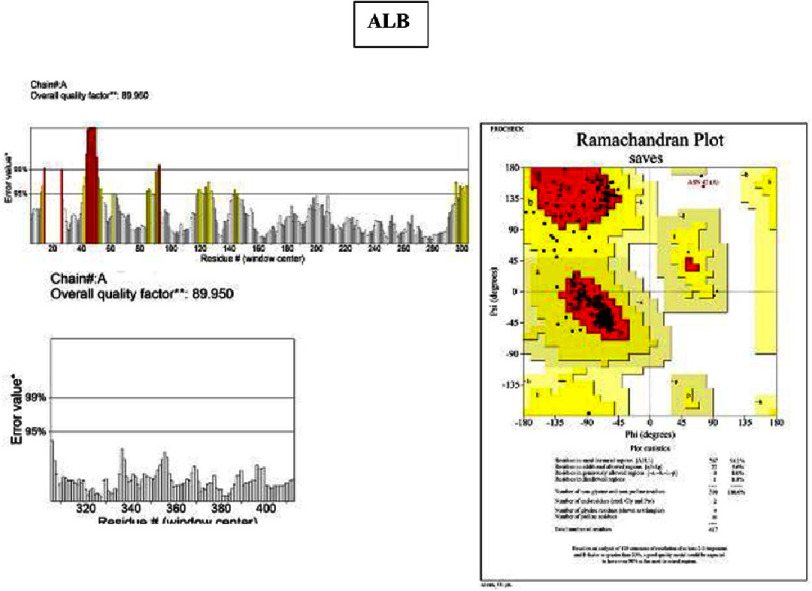
ERRAT-PROCHECK validation analysis. Analysis using UCLA-DOE LAB—SAVES v6.1 tools. ERRAT analysis showed a standard overall quality factor value ALB (89.9%) indicating acceptable structural quality and suitability for further structural and functional analysis. A PROCHECK analysis of the Ramachandran plot revealed the high value of residues in the allowed region of the ALB (94.1%), indicating good stereochemical quality and structural stability of the protein model.

Models with ERRAT scores ≥ 90% (MPO, ALB, CXCL8, CRP) demonstrated high structural reliability, while PROCHECK values approaching or exceeding 90% (IL-6, ICAM1, MPO, ALB, CXCL8, CRP) indicated satisfactory stereochemical quality, supporting their suitability for downstream molecular docking and further structural-functional investigation (Figs. 6–9).

## Discussion

This study employed an *in silico* bioinformatics approach to identify candidate targets for further investigation involved in the coronary no-reflow (CNR) phenomenon. This approach enables systematic prioritization of candidate genes based on integrated biological data. CNR is a complex clinical condition arising from structural and functional injury to the coronary microcirculation, driven by four well-established mechanisms: distal atherothrombotic embolization, ischemic myocardial injury, reperfusion injury, and individual susceptibility to microvascular damage^9^. These mechanisms collectively contribute to increased infarct size, reduced myocardial viability, and a higher risk of heart failure and mortality^10^. Understanding the genetic determinants underlying these pathways is therefore essential for guiding targeted therapeutic strategies.

### Genetic contribution to no-reflow pathophysiology

Genetic variations influence several biological pathways related to platelet activation, coagulation, plaque stability, endothelial function, angiogenesis, and antioxidant defense. Dysregulation in these pathways may heighten vulnerability to distal embolization, exacerbate ischemic damage, and impair reperfusion outcomes.^11^ In this study, 79 genes associated with CNR were identified, and enrichment analyses demonstrated that several biological processes and molecular functions are strongly implicated in the development of no-reflow.

### Biological processes contributing to no-reflow

#### Blood vessel diameter maintenance

“Blood vessel diameter maintenance” emerged as the most significantly enriched biological process. This finding highlights the central role of microvascular tone regulation in determining post-reperfusion flow. Under normal physiology, coronary arterioles maintain autoregulatory control to stabilize capillary hydrostatic pressure (CHP)^12^. In CNR, exhausted autoregulation disrupts this mechanism, rendering arterioles unable to preserve adequate CHP. During ischemia, pericytes constrict to maintain perfusion pressure; however, sustained or excessive contraction leads to capillary narrowing and microvascular obstruction^13^. The involvement of eleven genes in this process underscores vascular tone regulation as a strategic therapeutic target for preventing pericyte-mediated microvascular constriction.

#### Blood coagulation

“Blood coagulation” was also identified as a key biological process contributing to CNR. Plaque rupture activates the coagulation cascade, leading to the formation of platelet-rich thrombi and aggregates of erythrocytes and fibrin. Distal embolization of these components into the microcirculation represents a major pathway of microvascular obstruction^14^. Fibrin deposition at the microvascular level further intensifies capillary plugging and endothelial injury. The identification of thirteen genes within this pathway reinforces the pivotal role of coagulation as candidate targets for further investigation, particularly in the setting of primary PCI.

### Molecular function: Antioxidant activity

The enrichment of molecular functions indicated that antioxidant activity plays a significant role in the pathophysiology of CNR. Inadequately controlled oxidative stress impairs vasodilation, promotes platelet aggregation, enhances leukocyte adhesion, and accelerates endothelial injury^15,16^. The involvement of six antioxidant-related genes supports the concept that modulation of oxidative stress may serve as a promising therapeutic approach to mitigate reperfusion-related microvascular damage.

### Gene–gene interactions and identification of essential hub genes

STRING network analysis demonstrated a highly interconnected PPI network consisting of 55 nodes and 75 edges, indicating coordinated biological activity rather than random interactions. Centrality analyses using CytoHubba revealed essential hub genes across different functional domains. MCC analysis identified **IL6, TNF, ICAM1, MMP9, and CXCL8** as core regulators within inflammatory and endothelial dysfunction pathways. Genes such as **EDN1**and**NOS3** contribute to vasomotor regulation, while **CRP, MPO, and ALB** reflect systemic inflammatory and oxidative stress responses. In parallel, MNC analysis identified **VWF**and**SELP** as key connectivity hubs involved in platelet activation and endothelial adhesion processes known to facilitate microvascular obstruction^17–23^.

These findings are consistent with clinical evidence demonstrating the association of IL-6, TNF-*α*, ICAM-1, MMP-9, and CXCL8 with larger no-reflow areas, impaired ventricular recovery, and worse outcomes in STEMI patients undergoing PCI. Notably, the identified hub genes are well-established mediators of inflammation and oxidative stress, several of which have been previously associated with coronary no-reflow and myocardial injury.

Therefore, the primary contribution of this study lies not in identifying novel genes, but in providing a systematic and reproducible in silico framework for prioritizing biologically relevant targets in CNR. This approach should be regarded as hypothesis-generating, offering a structured foundation for future experimental and clinical validation studies.

Although this study relied primarily on GeneCards, several of the identified hub genes (e.g., IL6, TNF, CRP, MPO) have been consistently reported in curated databases such as DisGeNET and OMIM as being associated with inflammatory and cardiovascular conditions. This consistency supports the biological relevance of our findings while acknowledging the limitations of database selection.

### Protein structure validation and therapeutic target potential

Structural validation using ERRAT and PROCHECK showed that MPO, ALB, CXCL8, and CRP exhibited high-quality structural and stereochemical properties. ERRAT evaluates crystalline structural reliability, whereas PROCHECK assesses bond lengths, angles, and conformational geometry^24^. The favorable scores obtained in this study suggest that these proteins possess structural stability suitable for ligand binding, supporting their suitability as candidate targets for further structural and functional investigation. These validated structures provide a foundation for future molecular docking and experimental studies aimed at exploring potential therapeutic modulation.

### Study limitations and future directions

While GeneCards provides a comprehensive platform for gene prioritization by integrating data from multiple sources, it relies on algorithmic ranking rather than disease-specific curation. Therefore, the identified genes should be interpreted as prioritized candidates rather than definitive disease-specific markers.

Future studies incorporating curated databases such as DisGeNET, OMIM, or transcriptomic datasets (e.g., GEO) are warranted to validate and refine these findings, which may further improve reproducibility and strengthen the biological relevance of the results.

## Conclusion

This study highlights the potential role of genetic factors in the coronary no-reflow phenomenon and provides a systematic in silico framework for prioritizing candidate genes. Four hub genes (MPO, ALB, CXCL8, and CRP) were identified as biologically relevant candidates that warrant further experimental and clinical validation. These findings should be interpreted as hypothesis-generating and may guide future translational research in CNR.

## Authors’ contributions

**Conceptualization:** Fadhlan Abdur Rahman, Suryono Suryono

**Methodology:** Fadhlan Abdur Rahman, Suryono Suryono

**Software:** Fadhlan Abdur Rahman

**Validation:** Suryono Suryono, Aditha Satria Maula, Pipiet Wulandari

**Formal Analysis:** Fadhlan Abdur Rahman

**Investigation:** Fadhlan Abdur Rahman

**Data Curation:** Fadhlan Abdur Rahman

**Writing-Original Draft Preparation:** Fadhlan Abdur Rahman

**Writing-Review & Editing:** Suryono Suryono, Aditha Satria Maula, Pipiet Wulandari

**Visualization:** Fadhlan Abdur Rahman

**Supervision:** Suryono Suryono, Aditha Satria Maula, Pipiet Wulandari

All authors reviewed and approved the final manuscript and agree to be accountable for all aspects of the work.
